# Substance abuse treatment client experience in an employed population: results of a client survey

**DOI:** 10.1186/1747-597X-7-4

**Published:** 2012-01-17

**Authors:** Elizabeth L Merrick, Sharon Reif, Deirdre Hiatt, Dominic Hodgkin, Constance M Horgan, Grant Ritter

**Affiliations:** 1 Institute for Behavioral Health, Heller School for Social Policy and Management, Brandeis University, 415 South Street, MS035, Waltham, MA 02454-9110, USA; 2 Health Net, 2370 Kerner Boulevard, Mail Stop: 909 02 05, San Rafael, CA 94901, USA

## Abstract

**Background:**

Understanding client perspectives on treatment is increasingly recognized as key to improving care. Yet information on the perceptions and experiences of workers with private insurance coverage who receive help for substance use conditions is relatively sparse, particularly in managed behavioral health care organization (MBHO) populations. Furthermore, the role of several factors including prior service use has not been fully explored.

**Methods:**

Employees covered by a large MBHO who had received substance abuse services in the past year were surveyed (146 respondents completed the telephone survey and self-reported service use).

**Results:**

The most common reasons for entering treatment were problems with health; home, family or friends; or work. Prior treatment users reported more reasons for entering treatment and more substance use-related work impairment. The majority of all respondents felt treatment helped a lot or some. One quarter reported getting less treatment than they felt they needed.

**Discussion and conclusions:**

Study findings point to the need to tailor treatment for prior service users and to recognize the role of work in treatment entry and outcomes. Perceived access issues may be present even among insured clients already in treatment.

## Background

Improving care for substance use conditions includes making health care more patient-centered and accessible, according to the Institute of Medicine [1]. Understanding client perspectives on treatment and its results is increasingly recognized as key to improving care.

Most private health plans in the United States contract with managed behavioral health care organizations (MBHOs) for specialty behavioral healthcare,[2] as do many employers. MBHOs are specialized vendors that typically offer provider networks and utilization management specifically for behavioral health. Yet, there is little information available on the perceptions and experience of employees with employer-sponsored health coverage who receive help for substance use conditions, particularly when covered by MBHOs. These may be quite different from a public-sector treatment population or mixed populations of working and non-working individuals. Furthermore, the role of prior service use and certain sociodemographic characteristics has not been fully explored in this group.

Numerous studies have examined specific aspects of the treatment experience, from reasons for entering care to outcomes. A substantial body of literature on help-seeking has examined factors associated with the decision to enter treatment among persons with substance use conditions. Studies have found that the social consequences of addiction predict treatment entry [3] and provide mixed evidence on health problems as a factor [4]. Several demographic factors such as younger age, and clinical factors such as greater severity, have been associated with worse outcomes [5,6]. We discuss findings on gender and marital status below. Other studies have examined patient satisfaction and its relationship to treatment outcome, also with mixed results. For example, one study found a positive relationship between satisfaction near time of discharge and better drug use outcomes [7], but another found no significant relationship between satisfaction and several outcomes [8]. Other research has focused specifically on work-related outcomes. For example, in a managed care population in California, productivity gains following substance abuse treatment were substantial [9]. However, few of these studies have examined the range of patient-reported experience from treatment entry to perceived outcomes in order to obtain a fuller overall picture.

In addition to examining a broader range of client-reported experience, there is also a need for a better understanding of how this experience may differ for certain subgroups of clients. Prior use of services is one of these factors. There are several ways that prior service use could be related to reasons for treatment entry, experience of treatment, and anticipated outcomes. Persons who previously have used substance abuse treatment services may be more skilled in getting the help they need from providers, and may have more realistic expectations of treatment and its potential outcomes. Prior service users may have a lower threshold for re-engering treatment since they might understand their substance use issues as part of a long-term recovery process. On the other hand, to the extent that experienced treatment users are likely to have more problems, greater severity or a longer history of substance use problems, they may encounter greater difficulty in treatment and be less likely to achieve a successful outcome. They may also experience more discouragement, or have negative prior treatment experiences. In any case, it is useful to describe and compare the experiences of prior treatment users to those in treatment for the first time.

Previous studies have found that clients with more prior treatment episodes (or any prior treatment) generally had a greater level of problems than clients with fewer treatments [10-12]. However, one study found that treatment acceptance was greater for clients with more prior treatment, and both experienced and inexperienced clients made positive changes in multiple domains [10]. Long-term outcomes for clients with successful short-term outcomes have been found to be better for treatment-experienced than for treatment-naïve clients [13]

Studies on this topic have typically included the full range of clients found in publicly funded and other treatment programs. However, it is possible that these relationships could be different among persons who are employed and have private insurance coverage. Not as much is known about substance abuse treatment clients with stable employment, however there is some evidence that work outcomes such as absenteeism are improved [14].

Gender and marital status are among the multiple sociodemographic characteristics that have been investigated in relation to treatment entry, participation and outcomes. A review concluded that the evidence suggests women with substance use disorders are less likely than men, on a lifetime basis, to enter treatment [15] Gender was not found to be a significant predictor of treatment retention, completion, or outcomes. Gender-specific predictors of outcome do exist, however, and individual characteristics and treatment approaches can differentially affect outcomes by gender. [15.] Women may face particular barriers in treatment entry, including those related to program and treatment characteristics [16]. Furthermore, women may have different reasons for entering treatment and a different perspective on care received.

Marital status may also be important, given the potential for increased psychosocial and financial support. Being married has been found to be a protective factor against substance abuse, and also a predictor of less substance use during and after treatment [17]. Non-married status has been associated with symptom exacerbation during or shortly after treatment [18]. However, a systematic review of predictors of alcohol treatment outcomes found that marital status was not a consistent predictor [6] and a meta-analysis found that being married was not a strong predictor [19]. Most studies have either not focused primarily on employed, insured clients or did not differentiate between this and other types of populations. Thus, describing how the range of client experience may vary by marital status in an employed, privately insured group could be useful.

The goal of the current study was to add to knowledge in this area by surveying employees within a privately insured, MBHO-covered population who had used substance abuse treatment in the past year. The research questions were:

1. What are reasons for substance abuse treatment entry among privately insured, employed clients in an MBHO?

2. What are these clients' perceptions of treatment services, including barriers and work-related outcomes of care?

3. How do these vary by prior use of substance abuse treatment, as well as selected demographic characteristics?

## Methods

### Study Setting

The study setting was MHN, a national MBHO covering 11 million members for a range of services and products. MHN contracts with employers, health plans and other payers to manage and deliver specialty managed behavioral health and employee assistance program (EAP) services. This analysis focused on enrollees in MHN's commercial behavioral health and integrated behavioral health/EAP products. Enrollees seeking services contact MHN's call center and are referred to a provider. Benefits for treatment of substance use conditions vary by employer but typically cover outpatient, intermediate and higher levels of care.

### Data Sources

The primary data source is a telephone survey of employees covered by MHN's behavioral healthcare products who reported using substance abuse services of any type in the past 12 months. The sampling approach and survey response are described below. The survey was conducted from August 2009 through April 2010. The Survey Research Center at the University of Massachusetts-Boston conducted the telephone interviews. In addition, administrative data including claims and eligibility files were used for the purpose of sampling and also for non-response analysis. The study received IRB approval at the authors' institution and at the survey firm conducting the interviews. Subjects were provided with detailed information about the study through advanced letters as well as in the telephone survey script. Informed consent was obtained prior to beginning interviews. Each respondent received $20 for survey completion.

### Sample

**Figure 1:**
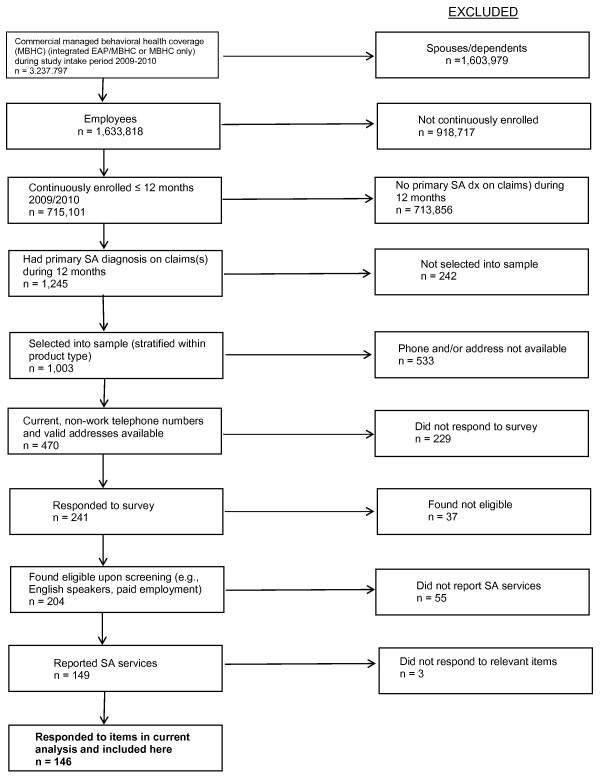
**Sampling Approach**.

Figure 1 shows the sampling approach for the study. The sample for this analysis (n = 146) is a subsample of a larger survey sample. These 146 subjects were among the 1,245 employees aged 18 or older who were covered by a managed behavioral health care product through MHN for at least 12 consecutive months during 2009 or 2010, and who also had a claim with a primary substance use condition diagnosis during those 12 months. All had comprehensive behavioral health benefits through MHN. From the 1,245 employees, a sample of 1,003 employees was randomly selected to be surveyed in a stratified design based on administrative data that indicated product type. The sampling was stratified by product type (managed behavioral health care through employer or health plan contracts, and integrated product incorporating employee assistance program [EAP] benefits) in order to achieve a more even mix of product coverage. Current, non-work (home or cell) telephone numbers were available for 470 employees. Current phone numbers were not available for the remainder of sample for several possible reasons, including that many employers do not require regularly updated home telephone numbers. Of those with an available current phone number, 241 responded to the survey and 204 completed screening and were found eligible. Screening eligibility criteria included paid employment during the past 12 months and ability to speak English. The response rate among persons who could be located by telephone was 50.6% based on an estimated eligibility rate of 84.5% among those who did not complete screening.

This study was part of a larger survey effort that included non-users of substance abuse treatment services. For reasons related to privacy and feasibility, the survey was designed so that interviewers did not know the treatment utilization status of subjects and each subject was asked about their utilization. Of the 204 eligible respondents, 149 reported receiving substance abuse treatment services in the past 12 months and were queried further about their service use; 146 answered items in the current analysis and are included here. It is possible that those who did not self-report service use did not consider their treatment to be primarily for substance use issues, did not wish to report it due to concerns about stigma or confidentiality, had recall errors in terms of precise timing, or did not disclose for other reasons.

Survey respondents did not differ significantly from non-respondents in the sample of 1,003 employees in terms of gender or region of residence, but were significantly older (mean 45.1 years with standard deviation [SD] 9.6 versus 42.7 years, SD 10.5, t = 2.9, DF = 961 p < .01). We also compared the 146 survey respondents in this analysis to all others among the 1,245 employees from which the sample was selected. We found that survey respondents were significantly older (mean 46.2 years, SD 9.7 compared to 43.4 years, SD 10.8; t = 2.93, DF = 1,243, p < .01). They did not differ significantly by gender or region.

### Survey Content

The survey content included respondents' reasons for accessing substance abuse treatment services, experience of care, and prior use of treatment. Respondents were asked about all services they used to get help with alcohol or drug use problems, including all settings and payment sources. Some items -- such as reasons for treatment entry and sources of encouragement or pressure to enter treatment -- focused specifically on the initial substance abuse treatment service used during the prior 12 months. Nearly all respondents in this analysis (144 out of 146) cited specialty treatment settings as their initial service (as opposed to general medical outpatient providers). Of the 146 respondents in this analysis, 138 indicated their initial service was paid for at least partly through employer-sponsored insurance or EAP, and 140 indicated that at least one type of substance abuse treatment service used during the year was paid for that way.

#### Substance abuse treatment services

Respondents were asked if they had, within the last 12 months, "used any services to get help with alcohol or drug use, such as going to a physician, substance abuse treatment program, a mental health or substance abuse professional" or if they had used EAP sessions to get help with "issues you had with alcohol or drug use". When more than one service was used, respondents were asked which occurred first and the treatment entry-related questions were focused on that service.

#### Treatment entry, experience of care, perceived barriers and outcomes

The survey included closed-ended questions about reasons for entering treatment. Respondents were asked to indicate for each of five possible reasons whether it was a reason they decided to obtain substance abuse treatment. In terms of sources of encouragement or pressure to enter treatment, four possible sources were asked about. Respondents were also asked whether to their knowledge there was communication between their substance abuse treatment provider and primary care physician, and how much they felt treatment has helped them (a lot, some, little, not all). Respondents were asked whether there was any time during the past year when they stopped treatment or receive less treatment than they wanted, and if so, asked whether each of five potential reasons for this perceived unmet need had occurred. The survey included questions about whether alcohol or drug issues had caused the respondent to miss work, or (separately) affected ability to perform work responsibilities when at work, before receiving substance abuse services during the past year. If respondents indicated such work impairment, they were asked whether the services received affected work attendance and job performance (better now, worse now, about the same as before).

#### Prior substance abuse treatment

Respondents were asked whether they had ever used services for alcohol or drug use issues before the past 12 months.

#### Sociodemographics and health

The survey contained items on gender, age, race, Hispanic ethnicity, marital status, education level, job status, supervisory role and health status.

### Statistical Analysis

Descriptive statistics are presented for sample description and survey items. Chi-square tests and t-tests were used for categorical and continuous variables respectively to conduct comparisons by prior treatment use, gender and marital status. For bivariate comparisons with small expected cell sizes weighted logistic regression was used. The data were weighted to account for probability of selection and non-response. Weights reflected the differential probability of selection for the different product types; enrollees in the integrated product were oversampled in order to obtain substantial representation.. In addition, the weights adjusted for nonresponse that varied slightly across product types. SAS Survey procedures were used to account for the survey design, with appropriate correction of standard errors.

## Results

### Client Characteristics

About two thirds of respondents were male and the large majority were between the ages of 35 and 54 (Table 1). Over 90% were white and a similar percentage were non-Hispanic. Married respondents accounted for 43.8% of the sample. Nearly one third had at least a four-year college education. About half (55.6%) of the sample reported being paid on an hourly basis, 30.9% were salaried, and 13.5% were currently unemployed although retaining employer-sponsored insurance coverage as the employee/subscriber. Most reported good to excellent health status, with only 11.3% reporting they were in fair or poor health. The majority (57.5%) had claims with an alcohol-related diagnosis, with no diagnoses of other substance use conditions. Most respondents were first-time treatment users. Type of substance abuse diagnosis noted on claims varied significantly by prior treatment use status, with an alcohol diagnosis alone being more common for first-time treatment users (66.8% versus 45.7% for experienced treatment users; chi-square = 6.2, degrees of freedom [DF] = 2, p < .05). There were no other statistically significant differences between those with versus without prior treatment experience. Most respondents (70.3%) reported using some level of care higher than standard outpatient office visits, with no significant difference between prior and new service users.

**Table 1:** Sample Description

		Weighted percent (standard error)		
	**Unweighted n**	**Total**	**Prior treatment users**	**First-time treatment users**

Unweighted N	146	100.0	62	84

Sex:				
Female	47	32.6 (3.9)	34.4 (6.1)	31.3 (5.1)
Male	99	67.4 (3.9)	65.5 (6.1)	68.7 (5.1)

Age:				
18-34	23	15.4 (3.0)	21.3 (5.3)	11.1 (3.4)
35- 44	39	26.6 (3.7)	24.1 (5.5)	28.4 (5.0)
45-54	63	43.5 (4.2)	42.2 (6.4)	44.4 (5.5)
55+	21	14.5 (3.0)	12.3 (4.2)	16.1 (4.1)

Race:				
White	137	93.6 (2.1)	96.6 (2.4)	91.4 (3.1)
African-American, Asian, other	9	6.4 (2.1)	3.4 (24)	8.6 (3.1)

Hispanic ethnicity	8	8.3 (2.0)	1.7 (1.7)	8.6 (3.1)

Marital Status:				
Married	63	43.8 (4.2)	38.4 (6.3)	47.7 (5.5)
Divorced/Separated/Widowed	44	30.6 (3.9)	31.9 (6.0)	29.7 (5.1)
Never married	38	25.6 (3.6)	29.7 (5.9)	22.6 (4.5)

Education level:				
HS or less	40	26.8 (3.7)	27.0 (5.7)	26.7 (4.9)
Some college	61	41.7 (4.0)	38.7 (6.3)	43.9 (5.4)
4-year college grad or more	45	31.5 (3.8)	34.3 (6.1)	29.4 (4.9)

Job status				
Salaried	45	30.9 (3.9)	35.9 (6.2)	27.2 (4.9)
Hourly	81	55.6 (4.2)	51.7 (6.5)	58.4 (5.4)
Reported not currently employed	20	13.5 (2.9)	12.3 (4.2)	14.4 (3.9)

Has supervisory role (if currently employed)	47	37.9 (4.4)	41.7 (6.8)	35.1 (5.7)

Health status:				
Excellent	18	12.3 (2.7)	10.1 (3.9)	14.0 (3.8)
Very good	51	35.2 (4.0)	34.9 (6.2)	35.4 (5.3)
Good	60	41.2 (4.1)	39.9 (6.3)	42.1 (5.4)
Fair/poor	17	11.3 (2.6)	15.1 (4.5)	8.6 (3.1)

Reported using higher level of care than regular outpatient	102	70.3 (3.8)	70.8 (5.9)	69.9 (4.9)

Any diagnosis on claims during year:			*	
Alcohol only	81	57.5 (4.2)	45.7 (6.4)	66.8 (5.4)
Drug only	38	28.4 (3.9)	37.2 (6.3)	21.6 (4.8)
Both	19	14.0 (3.0)	17.1 (4.9)	11.6 (3.6)

Note: Denominators for each cell vary slightly due to missing data; percents calculated from non-missing data. All variables missing < 2%. Chi-square tests used for all comparisons except Hispanic ethnicity for which logistic regression was used due to small cell sizes.

*p < .05

### Reported Reasons for Treatment Entry

Overall, the most common reasons reported for entering treatment were health concerns (65.4%), difficulties at home, with family or friends (62.8%), and difficulties at work (41.6%) (Table 2). Smaller proportions reported financial or legal concerns as reasons for entering treatment, or indicated none of the above. A significantly higher proportion of respondents with prior use of substance abuse treatment services cited difficulties at home, with family or friends (74.5% versus 54.3% of first-time service users; chi-square = 6.1, DF = 1, p < .05), difficulties at work (51.6% versus 34.2%, chi-square = 4.3, DF = 1, p < .05), and financial concerns (39.3% versus 23.5% chi-square = 4.3, DF = 1, p < .05). The mean number of reasons for treatment entry was 2.2 (standard error 0.1) and was significantly higher for persons with prior treatment compared to first-time service users (mean 2.5 versus 1.9, t = 3.1, DF = 144, p < .01). Examining reasons for entering treatment, women were significantly more likely than men to cite difficulties at home, with family or friends (75.9% versus. 56.4%, chi-square = 5.0, DF = 1, p < .05). There were no other significant differences by gender. Unmarried clients were more likely than married clients to cite work difficulties (51.7% versus 27.6%, chi-square = 8.2, DF = 1, p < .01), and were more likely to note financial difficulties as a reason for entering treatment (39.2% vs. 17.2%, chi-square = 8.3,, DF = 1, p < .01).

**Table 2:** Reported Reasons for Treatment Entry: Relationship with Prior Use of Service

	Weighted percent (standard error)		
**Reasons for entering treatment**	**Total**	**Prior treatment users**	**First-time treatment users**

Health concerns	65.4 (4.0)	69.3 (6.0)	62.6 (5.3)

Difficulties at home, with family or friends	62.8 (4.1)	74.5 (5.6)*	54.3 (5.5)

Difficulties at work	41.6 (4.2)	51.6 (6.5)*	34.2 (5.3)

Financial concerns	30.1 (3.9)	39.3 (6.3)*	23.5 (4.7)

Legal concerns	18.0 (3.2)	22.1 (5.3)	14.9 (3.9)

None of the above	7.3 (2.2)	4.5 (2.6)	9. 4 (3.2)

Total (row)	100.0	42.2 (4.1)	57.8 (4.1)

Note: Denominators for each cell vary slightly due to missing data; percents calculated from non-missing data. All variables missing < 2%. Chi-square tests used for all comparisons.

*p < .05

### Sources of Encouragement or Pressure to Enter Treatment

Just over half of the sample (51.6%) cited family or friends as a source of encouragement or pressure to enter treatment, followed by physician or other healthcare provider (24.4%) (Table 3). Much smaller numbers of respondents cited employer or supervisor, coworker, or EAP counselor. About one third did not indicate receiving encouragement or pressure to enter treatment from any of the five sources asked about. There were no statistically significant differences by prior use of treatment, gender or marital status.

**Table 3:** Sources of Encouragement or Pressure to Enter Treatment

	Weighted percent (standard error)		
**Received Encouragement/Pressure to Enter Treatment From:**	**Total**	**Prior treatment users**	**First-time treatment users**

Family or friends	51. 6 (4.2)	51.2 (6.5)	52.0 (5.5)

Physician or other health care provider	24.4 (3.6)	25.1 (5.6)	23.9 (4.7)

Employer or supervisor	13.3 (2.8)	10.7 (3.9)	15.2 (4.0)

Coworker	10.7 (2.6)	13.1 (4.4)	9.0 (3.1)

EAP counselor (if initial service was not EAP) n = 114	5.7 (2.1)	7.0 (3.8)	4.9 (2.4)

None of the above	33.5 (4.0)	35.3 (6.1)	32.1 (5.2)

Total (row)	100.0	42.0 (4.2)	58.0 (4.2)

All comparisons based on prior use of treatment are not statistically significant at p < .05. Chi-square tests used for all comparisons except EAP counselor for which logistic regression was used due to small expected cell sizes.

### Client Experience of Treatment and Perceived Barriers

Almost 85% of respondents indicated that treatment had helped either a lot (63.3%) or some (21.0%), with the remainder indicating they had been helped a little or not at all (Table 4). Among those with prior use of services, 72.9% indicated they had been helped a lot compared to 56.3% for those with no prior use, but this was not statistically significant (chi-square = 4.8, DF = 2, p = .09). Regarding coordination of specialty substance abuse and other medical care, only 18.4% of respondents indicated they believed that their specialty substance abuse treatment provider had communicated with their primary care physician. This did not vary significantly by prior use of services. It is likely that most of the respondents in this survey had a primary care physician, given their private insurance status and the fact that many were covered by health maintenance organization or preferred provider organization plans that require designation of a primary care physician. About one quarter (27.6%) of respondents indicated that treatment had stopped sooner or they had received less treatment than they wanted during the past year. The most common reason reported for this was problems with coverage or cost of services. These did not vary by prior use of services. There were no significant differences in experience of treatment and perceived barriers by gender. The only significant difference by marital status was that among those indicating perceived unmet need for treatment, married clients were more likely to cite coverage or cost as a reason (77.7% compared to 38.7%, chi-square = 6.4, DF = 1, p < .05).

**Table 4:** Client Experience of Care and Perceived Barriers

	Weighted percent (standard error)		
	**Total**	**Prior treatment users**	**First-time treatment users**

Client perception of how much treatment helped:			
A lot	63.3 (4.0)	72.9 (5.5)	56.3 (5.5)
Some	21.0 (3.3)	17.5 (4.6)	23.5 (4.7)
A little/not at all	15.7 (3.1)	9.6 (3.8)	20.2 (4.4)

Client believes SA provider communicated with PCP	18.4 (3.3)	15.2 (4.7)	20.8 (4.5)

Any times in past year when client stopped treatment sooner or received less treatment than wanted	27.6 (3.7)	34.0 (6.1)	23.0 (4.6)

(if so) Client-reported reasons:			
Treatment was not working	19.8 (6.4)	28.3 (10.1)	10.7 (7.2)
Time or location inconvenient	23.2 (6.7)	30.0 (10.1)	16.0 (8.6)
Problems with coverage or cost	55.2 (8.2)	53.5 (11.0)	57.0 (11.3)
Treatment staff were insensitive to cultural background (e.g., race, ethnicity, religion, age, sexual orientation, language preference)	2.7 (2.7)	0.0	5.5 (5.3)
Other problems with service provider	19.0 (6.1)	13.3 (7.5)	25.1 (9.2)

All comparisons based on prior use of treatment are not statistically significant at p < .05. Chi-square tests used for all comparisons.

### Perceived Work Impairment and Self-Reported Outcomes of Treatment

Nearly half of respondents indicated that before they received substance abuse services during the past 12 months, alcohol or drug problems had caused them to miss work (Table 5). A higher proportion of respondents with prior use of treatment reported this, compared to those with no prior use of treatment (58.4% versus 36.3%, chi-square = 6.9, DF = 1, p < .01). Among those who had missed work, the large majority (86.4%) indicated that the services they received resulted in better work attendance now. This did not differ significantly by prior use of treatment. Effects of alcohol or drug use on ability to perform work responsibilities when at work were reported by 42.9% of respondents, with a significantly higher proportion of those with prior treatment use reporting this (52.8% versus 35.8%, chi-square = 4.1, DF = 1, p < .05). Nearly all of these respondents (95.6%) reported that services they received had improved their ability to perform work responsibilities when at work. There was no significant difference in this based on prior use of treatment or marital status. A significantly higher proportion of women than men indicated that their work attendance had improved (97.2% versus 80.0%, chi-square = 5.3, DF = 1, p < .05).

**Table 5:** Perceived Work Impairment and Outcomes of Treatment

	Weighted percent (standard error)		
	**Total**	**Prior treatment users**	**First-time treatment users**

Alcohol/drug problems caused to miss work	45.6 (4.2)	58.4 (6.3)**	36.3 (5.3)

(If so) How have services affected work attendance?			
Attendance better now	86.4 (4.3)	89.9 (4.8)	82.4 (7.3)
About same	13.6 (4.3)	10.1 (4.8)	17.6 (7.3)
Worse now	0.0	0.0	0.0

Alcohol/drug problems affected work performance when at work	42.9 (4.2)	52.8 (6.5)*	35.8 (5.3)

(If so) How have services affected ability to perform work responsibilities when at work?			
Better now	95.6 (2.6)	96.8 (3.2)	94.3 (4.1)
About the same	4.4 (2.6)	3.2 (3.2)	5.7 (4.1)
Worse now	0.0	0.0	0.0

(If work performance was affected)			
Change in substance use --now compared to before receiving services during past year:			
Decreased	91.7 (3.6)	93.6 (4.4)	89.7 (5.6)
About the same	1.7 (1.7)	0.0	3.4 (3.4)
Increased	6.7 (3.2)	6.4 (4.4)	6.9 (4.7)

Chi-square tests used for all comparisons.

*p < .05 **p < .01

Respondents reporting impaired work performance were asked about changes in current substance use compared to before they received these services during the past year. The vast majority (91.7%) indicated that their substance use had decreased, with the remainder indicating that it had stayed about the same or increased. There was no statistically significant difference by prior use of treatment.

## Discussion

We discuss the findings in terms of topic addressed as well as comparisons by prior treatment use, gender or marital status. In this group of privately insured employees, the most commonly cited reasons for entering treatment were health concerns, difficulties at home, with family or friends, and difficulties at work. Although more than 41% indicated difficulties at work as a reason for entering treatment, far fewer indicated that they had received pressure or encouragement from work-related sources. This suggests that work-related coercion or encouragement, though evident for many individuals, is much less common than employees' perceptions of work problems as a factor in decision-making. It is possible that work-related problems were noticed and commented upon by others outside of the workplace, such as family members realizing that work problems stem from a substance use condition and urging treatment entry. It is also possible that for many people, work-related problems were not severe enough to provoke direct workplace involvement.

Respondents who reported prior use of substance abuse treatment were significantly more likely than first-time service users to report difficulties at home, with family or friends, financial concerns, and difficulties of work as reasons for treatment entry, and were similar in terms of other possible reasons. Experienced treatment users arrive in treatment with a greater burden of problems that precipitated treatment entry. They may need, be more likely to need, concrete help with financial and work problems as well as assistance with family and relationship issues related to substance use issues. Legal issues are present in this employed sample but were cited by less than one fifth of respondents.

Interestingly, women cited difficulties at home, with family or friends much more frequently as a reason for entering treatment than men. This may underline the particular importance of these relationships for women, and highlights the centrality of attending to the social system in treatment. The precise nature of the social connections, of course, is critical and may either support or hinder recovery. Our data do not address this issue but do disproportionately find difficulties in these relationships as a trigger for women's treatment seeking. The fact that unmarried clients were more likely than married clients to cite work in financial difficulties as a reason for entering treatment may reflect the potentially stabilizing influence of marriage-though as cited earlier, the literature has been mixed on this point.

There has been increasing concern about missed opportunities for identifying substance use conditions in medical settings and about the lack of integration or coordination between specialty behavioral health and general medical care, and there is hope that federal health reform will foster solutions to these problems [20]. The study findings illustrate the challenge of this aspect of health care. Although nearly two thirds of the respondents cited health concerns as a reason they entered treatment, only 24.4% indicated that a physician or other health care provider had encouraged or pressured them to enter treatment. It is possible that with better screening and intervention, patients might have received help sooner. The finding that only 18.4% of respondents believed their specialty substance abuse treatment provider communicated with their primary care physician also raises a red flag. This suggests a major lack of direct communication among providers, even if the clients themselves may have relayed information about their treatment to both types of providers. All of, these findings underline the ongoing need for better behavioral health and general medical care coordination, an important goal for the field [21].

Family and friends were identified as the number one source of encouragement or pressure to enter treatment. This supports findings of prior research [3,22]. About one third of the sample did not endorse any of the sources of encouragement or pressure that were asked about. It is likely that some of these might have received pressure from the legal/criminal justice system, while others in this group might not have received any specific encouragement or pressure to enter treatment.

It is interesting that despite the greater proportion of persons with prior treatment reporting several precipitating problems, there was no significant difference in the sources of encouragement or pressure. Thus it appears that persons with a treatment history are more likely to experience a number of precipitating problems in a subsequent treatment experience but are not more likely to be subject to pressure or encouragement. This seems surprising, but one possible explanation is that individuals with a treatment history are better able to recognize when they need help.

Most respondents found treatment helpful, and this was the case across prior service use, gender and marital status subgroups with no statistically significant differences. The apparently large difference in the helpfulness ratings of clients with versus without prior service use was not statistically significant. With the p value of less than .10 in this relatively small sample, however, this question merits further investigation in large samples. Clients with prior experience of treatment systems might have a better idea how treatment works and how to get the most out of it, they may feel more positively knowing that it has perhaps helped in the past, and they may have more realistic expectations regarding treatment.

About one quarter of respondents in this privately-insured group reported stopping treatment sooner or getting less treatment than they wanted. Many of these respondents felt that treatment was not working, that treatment time or location was inconvenient, or had other problems with service providers. These are helpful findings for providers seeking to better engage their clients. Half of the respondents reporting unmet need cited problems with coverage or costs as the reason. According to the National Household Survey on Drug Use and Health, the vast majority of people with unmet need for substance abuse treatment fail to recognize that they have a problem [23]. However, among those who do recognize their problem and make an effort to get help, 9% indicate that they have health coverage but it did not cover treatment or cover the costs. The findings from the study presented here offer insight into the viewpoints of those with comprehensive, employer-sponsored coverage who have successfully accessed treatment. Without detailed clinical data it is impossible to ascertain whether further treatment was clinically indicated. Future work might specifically focus on this issue in a larger sample, and examine detailed treatment patterns in relation to perceived barriers. Another potential barrier, lack of sensitivity to cultural background such as race, ethnicity, religion, age, sexual orientation, or language preferences, proved to be rare in practice within this sample.

Reported work impairments were common, supporting prior evidence [24]. The vast majority of respondents felt that treatment had improved work attendance and ability to perform responsibilities when at work, as well as helping to decrease substance use. The finding that prior service users were significantly more likely to report work impairments is similar to the findings that higher proportions of prior service users endorsed various problem areas as reasons for entering treatment. In this context, it is interesting, and heartening, that both experienced and first-time service users reported these positive outcomes in similar proportions. The fact that women were more likely than men to indicate that their work attendance had improved as a result of services received provides an interesting nuance, given that they had similar global ratings of how much treatment had helped.

There are several limitations to the study. The sample was drawn from a single MBHO's covered population, thus generalizability may be limited to the extent that treatment arrangements vary by MBHO. However, MHN's benefits are generally comprehensive in terms of a continuum of care and carry benefit levels similar to many other MBHOs. Generalizability is also limited by the demographics of the sample, including the small numbers of nonwhite or young employees. Another limitation is loss of sample due to unavailability of home telephone numbers, and substantial non-response. The relatively small sample size means that there was limited statistical power for some conditional analyses, although numerous observed differences did attain statistical significance. The study nonetheless provides a rare window into the experience of employed clients in substance abuse treatment through an MBHO, which can also inform future studies. This is similar to other studies with small samples that can illuminate under-studied topics [25]. The measures derived from survey items have not been validated. These analyses and comparisons of experienced and first-time treatment users are essentially descriptive in nature. Future work could examine in more depth selected experience of care or outcomes measures in a multivariate context, to control for multiple confounders such as the fact that new users were more likely to have alcohol-only diagnoses. Detailed clinical data on factors such as severity of substance use conditions were not available. Respondents received different types and quantities of services so this study is a snapshot of clients recently in some form of treatment. Not all of the services received were specialty treatment services provided by MHN. However, the vast majority of services reported on were specialty substance abuse treatment services provided through respondents' employer-sponsored benefits, indicating MHN services. Despite these limitations, these data offer further insight into perceptions of substance abuse treatment in a privately insured, largely working population.

## Conclusions

The findings of this study provide a useful picture of substance abuse treatment users who are (or were recently) employed and have employer-sponsored private insurance coverage. Characterizing this sample of service users who are covered by a large managed behavioral healthcare organization is helpful in understanding who is accessing care, how employees decide to enter treatment, and how they experience care and perceive treatment outcomes. These clients often initiate treatment with multiple concerns that precipitated entry, and in this sample, those with prior treatment use reported more problems. Women were more likely to report difficulties at home, with family or friends as a reason for entering treatment. MBHO's and treatment providers can use these findings to enhance their approach to addressing multiple concerns and difficulties in relationships-especially for women-tailoring treatment to be as client-centered as possible.

Receiving pressure or encouragement from health care or work-related sources reportedly happens much less frequently than the existence of relevant precipitants such as health concerns or work issues. The findings suggest room for improvement in identification of substance use conditions and treatment facilitation within health care and employment settings. Wider adoption of screening, brief intervention and referral to treatment (SBIRT) in primary care settings is effective for alcohol use and shows promise for other substances as well [26,27]. Employers can heighten their efforts to improve early identification and access through mechanisms including employee assistance programs which can incorporate specific methods such as SBIRT [24,28].

The fact that one quarter of this sample of people with insurance coverage (including for specialty substance abuse treatment), who had received some treatment, still felt that treatment had been cut short or they did not receive as much treatment as they needed indicates that the perception of unmet need can occur even among the relatively well insured. Further research on this particular aspect would be useful in order to obtain more detailed information including provider and health plan perspectives on these situations.

The study findings, taken together, will be useful in systematic treatment planning through providing a picture of motivating factors and need areas and unemployed, privately insured group with MBHO coverage. They will also inform efforts to improve access and coordination of care that take into account the current patterns of encouragement, pressure, and cross-sector communication from the client perspective.

## List of abbreviations used

MBHO: managed behavioral health care organization; EAP: employee assistance program.

## Competing interests

Dr. Hiatt is employed at Health Net, the parent corporation for MHN, and was previously employed at MHN. The other authors have no competing interests to declare.

## Authors' contributions

ELM led the study design, data collection, analysis and drafting of the manuscript. SR and DH (Hiatt) participated in analysis, interpretation, and manuscript revisions. DH (Hodgkin) and CH contributed to interpretation of findings and manuscript revisions. GR provided statistical consultation. All authors read and approved the final manuscript.
